# The spectral signature of cloud spatial structure in shortwave irradiance

**DOI:** 10.5194/acp-16-13791-2016

**Published:** 2016-09-28

**Authors:** Shi Song, K. Sebastian Schmidt, Peter Pilewskie, Michael D. King, Andrew K. Heidinger, Andi Walther, Hironobu Iwabuchi, Gala Wind, Odele M. Coddington

**Affiliations:** 1 Department of Atmospheric and Oceanic Sciences, University of Colorado, Boulder, CO, USA; 2 Laboratory for Atmospheric and Space Physics, University of Colorado, Boulder, CO, USA; 3 NOAA Center for Satellite Applications and Research, Madison, WI, USA; 4 Center for Atmospheric and Oceanic Studies, Tohoku University, Sendai, Japan; 5 Space Systems and Applications, INC., Greenbelt, MD, USA

## Abstract

In this paper, we used cloud imagery from a NASA field experiment in conjunction with three-dimensional radiative transfer calculations to show that cloud spatial structure manifests itself as a spectral signature in shortwave irradiance fields – specifically in transmittance and net horizontal photon transport in the visible and near-ultraviolet wavelength range. We found a robust correlation between the magnitude of net horizontal photon transport (*H*) and its spectral dependence (slope), which is scale-invariant and holds for the entire pixel population of a domain. This was surprising at first given the large degree of spatial inhomogeneity. We prove that the underlying physical mechanism for this phenomenon is molecular scattering in conjunction with cloud spatial structure. On this basis, we developed a simple parameterization through a single parameter *ε*, which quantifies the characteristic spectral signature of spatial inhomogeneities. In the case we studied, neglecting net horizontal photon transport leads to a local transmittance bias of ±12–19 %, even at the relatively coarse spatial resolution of 20 km. Since three-dimensional effects depend on the spatial context of a given pixel in a nontrivial way, the spectral dimension of this problem may emerge as the starting point for future bias corrections.

## 1 Introduction

Determining cloud radiative effects for scenes with a high degree of spatial complexity remains one of the most persistent problems in atmospheric radiation, especially at the surface where satellite observations can only be used indirectly to infer energy budget terms. In the shortwave (solar) spectral range, it is especially challenging to derive consistent albedo, absorption, and transmittance from spaceborne, aircraft, and ground-based observations for inhomogeneous cloud conditions (Kato et al., 2013; Ham et al., 2014). This problem is closely related to the long-debated discrepancy between observed and modeled cloud absorption (Stephens et al., 1990) since energy conservation for a three-dimensional (3-D) atmosphere (Marshak and Davis, 2005, Eq. 12.13),

(1) R+T=1−(A+H),

connects reflectance *R*, transmittance *T*, and absorptance *A* of a layer. The term *H* accounts for lateral net radiative flux from pixel to pixel (which we will call net horizontal photon transport). Out of necessity, most algorithms for deriving *R, T*, and *A* from passive imagery inherently presume isolated pixels by relying on 1-D radiative transfer (independent pixel approximation), which does not reproduce *H*. Net horizontal photon transport has therefore long been a common explanation not only for inconsistencies between measured and calculated broadband cloud absorption (Fritz and MacDonald, 1951; Ackerman and Cox, 1981) but also for remote sensing artifacts (Platnick, 2001).

Observational evidence for this explanation emerged with the availability of spectrally resolved aircraft measurements of shortwave irradiance (Solar Spectral Flux Radiometer, SSFR: Pilewskie et al., 2003). Schmidt et al. (2010) derived apparent absorption, the sum of *A* and *H*, from irradiance measurements aboard the NASA ER-2 and DC-8 aircraft that flew along a collocated path above and below a heterogeneous anvil cloud during the Tropical Composition, Cloud and Climate Coupling Experiment (TC^4^) (Toon et al., 2010). The results of this study showed that, in absolute terms, *H* at visible wavelengths (where cloud and gas absorption are negligible) can attain a similar magnitude as the absorbed irradiance *A* at near-infrared wavelengths. Horizontal photon transport thus has the potential to mimic substantially enhanced absorption. Three-dimensional calculations confirmed the measurements, and radiative closure was achieved within measurement and model uncertainties without invoking proposed enhanced gas absorption (Arking, 1999) or big cloud droplets (Wiscombe et al., 1984). The results also suggested that the overestimation of absorption would persist even when averaging over long distances as proposed by Titov (1998). This is simply because radiation flight legs are often preferentially targeted at cloudy regions (〈*H*〉 > 0) and do not adequately sample clear-sky areas where photons are depleted (〈*H*〉 < 0), which is interpreted as apparent emission in measurements.

Perhaps the most significant finding by Schmidt et al. (2010) was the distinct spectral shape of *H* from the near-ultraviolet well into the visible wavelength range, leading to the notion of “colored” net horizontal photon transport (Schmidt et al., 2014). A previous study addressing horizontal photon transport from an energy budget point of view (Kassianov and Kogan, 2002) had focused on the wavelength range of 0.7–2.7 µm, specifically to avoid molecular scattering at shorter wavelengths. Strategies for mitigating the overestimation of cloud absorption (Ackerman and Cox, 1981; Marshak et al., 1999) require that *H* be more or less constant in the visible wavelength range (Welch et al., 1980), and so the discovery of the spectral dependence of *H* suggested that they should be applied with caution. For example, Marshak et al. (1999) in their conditional sampling technique required that *H* = 0 for at least two different wavelengths. Kindel et al. (2011) applied such a modified scheme for boundary layer clouds.

Further analysis of the relationship between cloud structure and its spectral signature, presented here, revealed a surprisingly robust correlation between the magnitude of *H* and its spectral slope, d*H* / d*λ*. In the course of this paper, we provide evidence for molecular scattering as the physical mechanism behind this correlation, and develop a simple parameterization based on this knowledge. We also examined at which spatial aggregation scale *H* can be ignored and whether the correlation between *H* and d*H* / d*λ* is scale-invariant. Finally, we considered the ramifications of our findings on the shortwave surface energy budget.

Following this introduction, we provide definitions of relevant terms and explain how *H* relates to top-of-atmosphere (TOA) and surface cloud radiative effects (CREs). We then discuss the data and model calculations that lay the basis for our study (Sects. 3 and 4). In Sect. 5, we discuss the correlations between *H* and d*H* / d*λ*, followed by the underlying physical mechanism and parameterization presented in Sect. 6. The discovered relationship is then examined as a function of spatial scale (Sect. 7) and interpreted in terms of the surface CREs (Sect. 8). In the conclusions, we discuss the significance of our findings and propose multispectral or spectral techniques for deriving first-order correction factors in CRE estimates from space, aircraft, and from the surface that may render 3-D calculations unnecessary.

## 2 Net horizontal photon transport and cloud radiative effect

The instantaneous radiative effect of any atmospheric constituent is the difference of net irradiance (flux density) in its presence (all-sky) and absence (clear-sky). For clouds, we define

(2) $${\text{CRE}}_{\lambda }=\;[\frac{{\left(F_{\lambda }^{↓}-F_{\lambda }^{↑}\right)}_{\text{all}-\text{sky}}}{F_{\lambda }^{↓,\text{ TOA}}}-\frac{{\left(F_{\lambda }^{↓}-F_{\lambda }^{↑}\right)}_{\text{clear}-\text{sky}}}{F_{\lambda }^{↓,\text{ TOA}}}]\times 100\%,$$

where $F_{\lambda }^{↓}$ and $F_{\lambda }^{↑}$ are downwelling and upwelling irradiance, and their difference is net irradiance. For this paper, we normalize the absolute radiative effect by the TOA downwelling irradiance $\left(F_{\lambda }^{↓,\text{TOA}}\right)$, and consider the relative radiative effect as percentage of the incident irradiance. We use spectrally resolved rather than broadband quantities, indicated by subscript *λ*.

The TOA shortwave CRE is always negative (cooling effect) because the reflected irradiance $F_{\lambda }^{↑,\text{TOA}}$ in the presence of clouds is larger than for clear-sky conditions. The surface shortwave CRE is also negative because clouds decrease the transmitted irradiance $F_{\lambda }^{↓,\text{SUR}}$, at least for homogeneous conditions; broken clouds can locally increase surface insolation. In contrast to the shortwave CRE at TOA and at the surface, clouds have a warming effect on the layer in which they reside. For homogeneous conditions (*H* = 0), this can be quantified in terms of the layer property absorptance

(3) $$A_{\lambda }=[\frac{F_{\lambda }^{↓,\text{ top}}-F_{\lambda }^{↑,\text{ top}}}{F_{\lambda }^{↓,\text{ top}}}-\frac{F_{\lambda }^{↓,\text{ base}}-F_{\lambda }^{↑,\text{ base}}}{F_{\lambda }^{↓,\text{ top}}}]\times 100\%,$$

for a cloud located between *h*_top_ and *h*_base_ with the same normalization as used above for the relative CRE. It can be determined from aircraft measurements by collocated legs above and below the cloud (Schmidt et al., 2010). The warming within the layer arises from absorption (*A* > 0) primarily in the near-infrared wavelength range (1 µm < *λ* < 4 µm). Similarly, layer transmittance and reflectance are defined as

(4) $$T_{\lambda }=(\frac{F_{\lambda }^{↓,\text{ base}}}{F_{\lambda }^{↓,\text{ top}}})\times 100\%$$

(5) $$\text{and }R_{\lambda }=(\frac{F_{\lambda }^{↑,\text{ top}}-F_{\lambda }^{↑,\text{ base}}}{F_{\lambda }^{↓,\text{ top}}})\times 100\%.$$

Related to layer reflectance is the albedo $\alpha_{\lambda }=F_{\lambda }^{↑}/F_{\lambda }^{↓}$. The sum of layer absorptance, transmittance, and reflectance defined in this way is 100 %, and thus satisfies energy conservation for horizontally homogeneous layers. For individual pixel sub-volumes within an inhomogeneous layer (voxels), *A_λ_* in Eq. (3) can be replaced with *A_λ_* + *H_λ_* ≡ *V_λ_*, where *V_λ_* stands for the vertical flux divergence (the net irradiance difference above and below a layer). In this way, energy conservation including horizontal transport (Eq. 1) is retained.

The difference of the CRE at TOA and at the surface from Eq. (2) can be related to Eq. (3) as follows:

(6a) $${\text{CRE}}^{\text{TOA}}-{\text{CRE}}^{\text{surface}}=\;[\frac{{\left(F_{\lambda }^{\text{net},\text{ cloud}}-F_{\lambda }^{\text{net},\text{ clear}}\right)}^{\text{TOA}}}{F_{\lambda }^{↓,\text{ TOA}}}\;-\frac{{\left(F_{\lambda }^{\text{net},\text{ cloud}}-F_{\lambda }^{\text{net},\text{ clear}}\right)}^{\text{surface}}}{F_{\lambda }^{↓,\text{ TOA}}}]\times 100\%$$

(6b) $$=[\frac{{\left(F_{\lambda }^{\text{net},\text{ TOA}}-F_{\lambda }^{\text{net},\text{ surface}}\right)}^{\text{cloud}}}{F_{\lambda }^{↓,\text{ TOA}}}\;-\frac{{\left(F_{\lambda }^{\text{net},\text{ TOA}}-F_{\lambda }^{\text{net},\text{ surface}}\right)}^{\text{clear}}}{F_{\lambda }^{↓,\text{ TOA}}}]\times 100\%.$$

The first term inside the brackets of Eq. (6b) is identical to *A_λ_* from Eq. (3) if the boundaries of the layer *h*_top_ and *h*_base_ are extended to the TOA and surface, respectively. We denote this by *Â_λ_* and distinguish full-column properties using a caret (*Â, Ĥ, R̂, T̂*) from the layer properties that bracket only the cloud itself (*A, H, R, T*). The second term in Eq. (6b) stems from clear-sky absorption by atmospheric constituents other than clouds (gases and aerosols).

Equation (6b) can then be rewritten as

(6c) $${\hat{A}}_{\lambda }={\text{CRE}}^{\text{TOA}}-{\text{CRE}}^{\text{surface}}\;+[\frac{{\left(F_{\lambda }^{\text{net},\text{ TOA}}-F_{\lambda }^{\text{net},\text{ surface}}\right)}^{\text{clear}}}{F_{\lambda }^{↓,\text{ TOA}}}]\times 100\%,$$

which simply means that the total atmospheric column absorption comprises contributions from the cloud itself as well as from clear-sky absorption. In the presence of horizontal inhomogeneities, the left and right side of Eq. (6c) may be inconsistent unless *Â_λ_* is replaced with *V̂_λ_* = *Â_λ_* + *Ĥ_λ_* as above.

Presented in this way, the central role of absorptance and horizontal transport in linking the net irradiances above and below a cloud (Eq. 3), as well as the TOA and surface CRE (Eq. 6c), becomes clear. While the global TOA CRE can directly be derived from reflected radiances (Loeb et al., 2005), for example from the Clouds and the Earth’s Radiant Energy System (CERES) on the Aqua and Terra satellites (Wielicki et al., 1996), the derivation of the surface CRE also requires the knowledge of atmospheric absorptance or transmittance. In the case of CERES, the required cloud properties are obtained from retrievals of the accompanying imager, the Moderate Resolution Imaging Spectroradiometer (MODIS) (Minnis et al., 2011). As stated in the previous section, this is accomplished through lookup tables which are based on 1-D calculations and therefore do not provide *H*.

Recognizing the crucial significance of horizontal photon transport for obtaining an accurate surface CRE, Barker et al. (2012) and Illingworth et al. (2015) described the ambitious goal of using 3-D radiative transport operationally in the European radiative budget experiment Earth Clouds, Aerosols and Radiation Explorer (EarthCARE). They tested their algorithm with A-Train data. As a metric for 3-D effects, they employed the commonly used difference between 3-D and IPA calculations (e.g., Scheirer and Macke, 2003). In a similar manner, Ham et al. (2014) calculated the effect of horizontal photon transport on cloud absorption, transmission, and reflected radiance. They found these three quantities to be correlated when stratifying their results by cloud type after spatial aggregation to at least 5 km.

Since the studies cited above pertained to EarthCARE and CERES, they only considered broadband effects. This does not allow the separation of *A_λ_* and *H_λ_* by means of their distinct spectral characteristics. Our approach, first presented by Schmidt et al. (2014), bridges this gap. In this paper, we focus exclusively on the near-ultraviolet and visible wavelength range, and explore the spectral fingerprint from cloud inhomogeneities in conjunction with molecular scattering in *H_λ_*, which also imprints itself on reflected radiances (Song, 2016; Song et al., 2016). We chose not to include aerosols in either study, primarily to isolate the spectral signature of heterogeneous clouds before considering the more general case of clouds and aerosols in combination.

## 3 Cloud data

Our study builds upon the results by Schmidt et al. (2010), and therefore uses the same cloud case, a tropical convective core with anvil outflow, observed during the TC^4^ experiment on 17 July 2007 (from 15:19 to 15:35 UTC) by the NASA ER-2 aircraft about 300 km south of Panama. Two realizations of the observed cloud field were used as input to 3-D radiative transfer calculations, one based on airborne imagery only (as in the earlier study, Sect. 3.1), and one based on merged airborne and geostationary imagery (Sect. 3.2) to study large-scale effects.

### 3.1 Sub-scene from ER-2 passive and active remote sensors

Level-2 cloud retrievals of the Moderate Resolution Imaging Spectrometer (MODIS) Airborne Simulator (MAS: King et al., 1996, 2010) were combined with reflectivity profiles from the Cloud Radar System (CRS: Li et al., 2004) as described in detail by Schmidt et al. (2010). The primary information originates from MAS optical thickness, thermodynamic phase, effective radius, and cloud top height retrievals for each pixel (*x,y*) within the imager’s swath (roughly 20 km for a cloud top height of 10 km). The imagery-derived information was extended into the vertical dimension *z* by simple approximations as follows.

The effective radius from MAS, *r*_e_ (*x,y*), was used throughout the vertical dimension *z* although only representative of the topmost layer. Since the study is limited to the near-ultraviolet and visible wavelength range where cloud absorption is negligible, this simplification only affects the scattering phase function. Approximating it with that at cloud top is acceptable because to first approximation, 3-D radiative transfer is determined by the distribution of cloud extinction.

The MAS-retrieved optical thickness *τ* (*x,y*) for each pixel was vertically distributed by using the water content (WC) profile from CRS: WC(*z*) = 0.137 × *Z*^0.64^ (Liu and Illingworth, 2000), where *Z* is the radar reflectivity from CRS in dBZ. Since WC(*z*) is only available along the flight track, nadir-only CRS profiles were also used across the entire MAS swath (shifted vertically by *z*_0_ to match the MAS cloud top height at off-nadir pixels). Cloud extinction *β* for each voxel (*x,y,z*) was thus obtained as *β* (*x,y,z*) = *τ*_MAS_ (*x,y*) × WC(*z* + *z*_0_)/Σ_*z*_WC(*z*). Along the flight track, the mismatch between MAS- and CRS-retrieved cloud top height is ≤ 0.5 km. The CRS-derived average cloud top height is 10.8 km, and the mean geometrical thickness is 3.3 km.

The resulting cloud field was gridded to a resolution of 0.5 km horizontally (similar to the MODIS pixel size of some channels) and 1.0 km vertically (chosen larger than the mismatch between CRS and MAS in cloud top height).

**Figure 1:**
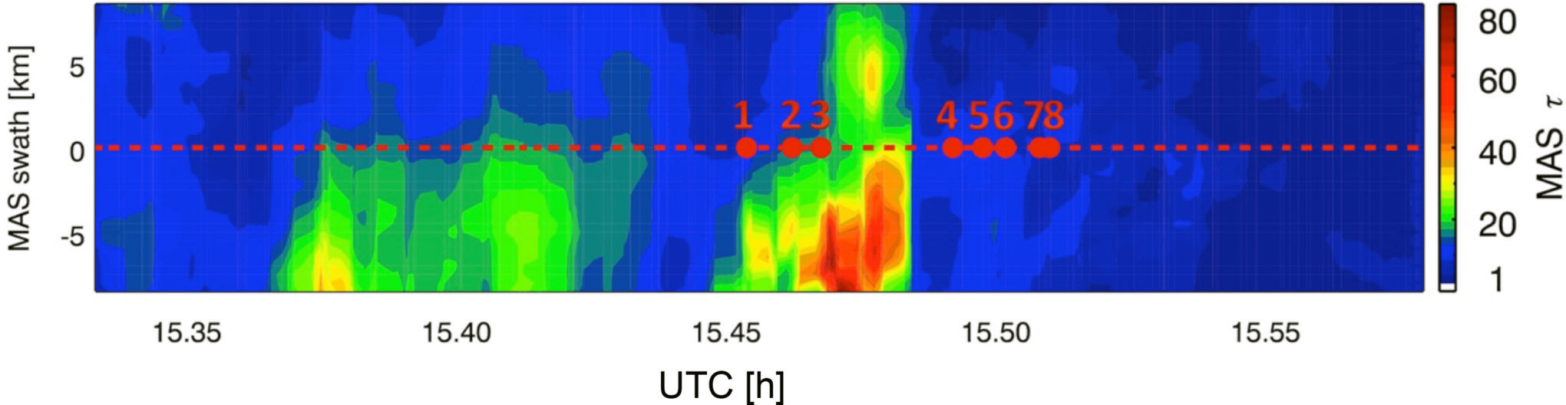
Cloud optical thickness from MAS along an ER-2 leg from 17 July 2007 (length: 192 km, swath: 17.5 km), re-gridded to a horizontal resolution of 500 mm. The red dashed line indicates the ER-2 flight track in the center of the MAS swath. Results of net horizontal photon transport for the eight highlighted pixels are shown in Table 1 and Fig. 3a.

Figure 1 shows the cloud optical thickness field from MAS after regridding, with the nadir track highlighted as a dashed line. The length of this scene is 192 km (384 pixels in *x*), and the width is 17.5 km (35 pixels in *y*).

### 3.2 Large-scale field from ER-2 data merged with geostationary imagery

To generalize our findings to larger scales than 17.5 km, we embedded the sub-scene from the ER-2 remote sensors in the context of the large-scale cloud field as retrieved from the Geostationary Operational Environmental Satellite East (GOES-12). The imager on board GOES-12 has five channels centered at 0.65, 3.9, 6.7, 10.7, and 12.0 µm. In the sampling region, cloud property retrievals were produced at 15:15 and 15:45 UTC (Walther and Heidinger, 2012; Heidinger et al., 2013). We chose the earlier time because it was more consistent with the MAS retrieval in terms of the optical thickness along the ER-2 track. There are small discrepancies between the GOES and MAS cloud top height retrievals, which are due to a combination of the different spatial resolutions, and channels that are used for the respective retrievals (Walther and Heidinger, 2012; Platnick et al., 2003; King et al., 2010). For the purpose of this study, these differences are not significant.

**Figure 2:**
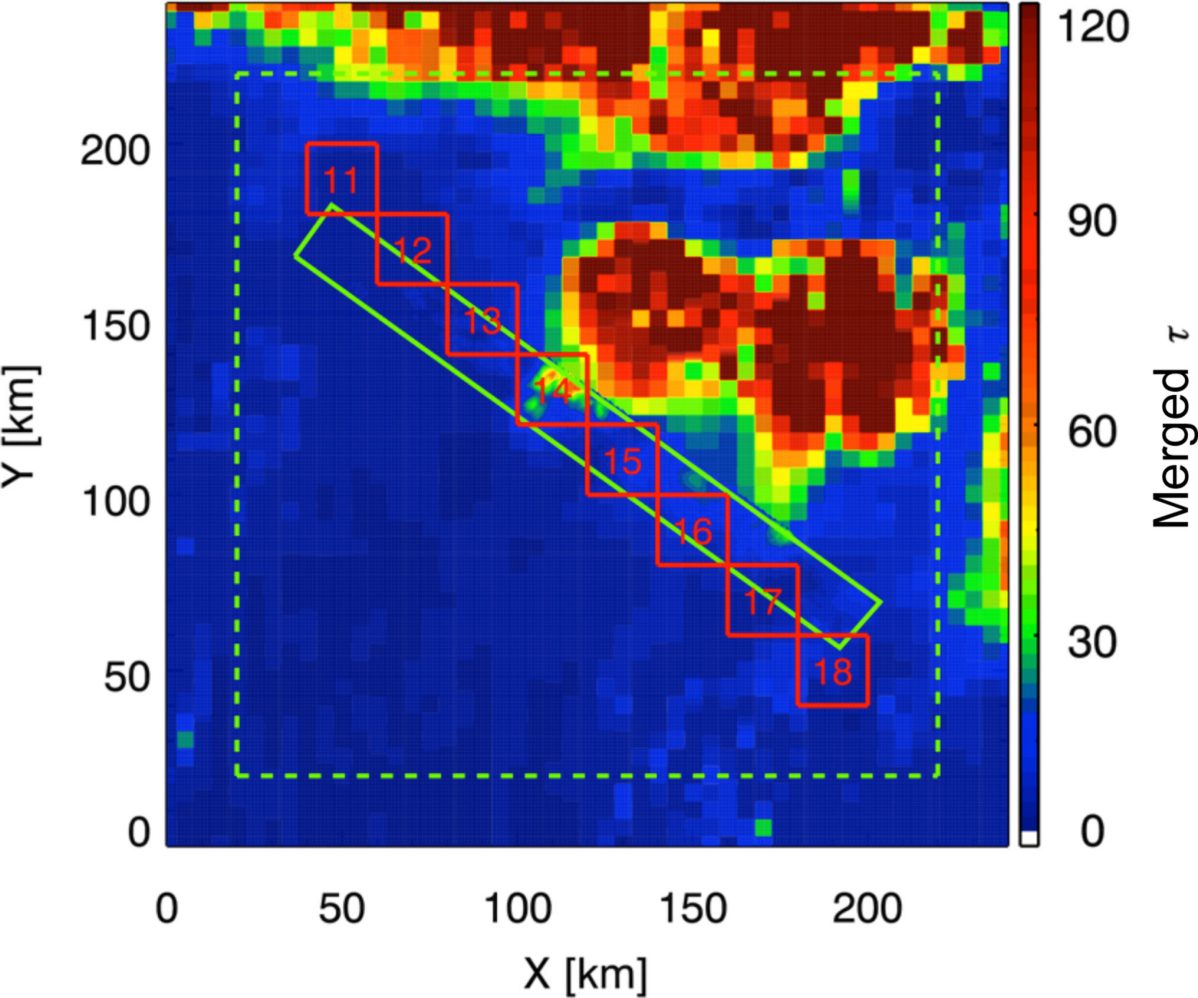
Optical thickness of the large-scale cloud field. The green rectangle marks the embedded MAS swath (Fig. 1); the red squares mark 20 km “super-pixels” within the scene. Radiative transfer model output outside the dashed green square is discarded (see Sect. 7).

Figure 2 shows the extended cloud scene (240 km × 240 km). Outside the MAS swath, GOES-12 retrievals were used instead of those from MAS. Similarly, as for the sub-scene cloud, the effective radius retrieval was extended throughout the vertical dimension. The optical thickness was distributed vertically using the CRS profile with the closest match in column-integrated water path (as compared to the retrieved value from GOES) and adjusted in altitude to match the cloud top height retrievals from GOES-12. This approach for distributing profile information from active instrumentation across the swath of a passive imager is more simplistic than that developed by Barker et al. (2011) who used multispectral radiances from MODIS. Transferring radar information to off-nadir pixels as far away as 120 km is not necessarily justified due to spatial decorrelation of cloud systems (Miller et al., 2014). However, in the absence of any other information, it was considered the best alternative to estimating the cloud vertical structure without any a priori knowledge.

## 4 Model calculations

The calculations in this study were performed with the 3-D Monte Carlo Atmospheric Radiative Transfer Simulator (MCARaTS: Iwabuchi, 2006). MCARaTS is an open-source code written in FORTRAN-90, which can be obtained at www.sites.google.com/site/mcarats/. It calculates shortwave and longwave spectral or broadband radiances and irradiances based on a forward propagating photon transport algorithm. It is optimized to run efficiently on parallel computers.

In addition to the two 3-D cloud fields described in Sect. 3, the standard tropical summer atmosphere as distributed within the libRadtran radiative transfer package (www.libradtran.org: Mayer and Kylling, 2005) was used to prescribe the vertical profile of temperature, pressure, water vapor, and other atmospheric gases. For gas molecular scattering, we calculated the optical thickness for each layer using the approximation by Bodhaine et al. (1999), and used the built-in Rayleigh scattering phase function from MCARaTS. For gas molecular absorption, we adopted the correlated *k*-distribution method described by Coddington et al. (2008). It was originally based on Mlawer and Clough (1997), modified for the shortwave by Bergstrom et al. (2003), and was specifically developed for the Solar Spectral Flux Radiometer (SSFR: Pilewskie et al., 2003). The SSFR instrument line shape (6–8 nm full-width half-maximum) defines the width of the channels in this study (narrower than MODIS or MAS channels). The spectrum by Kurucz (1992) served as the extraterrestrial solar spectrum.

Calculations were performed at 11 wavelengths ranging from the near ultraviolet to the very-near infrared (350, 400, 450, 500, 550, 600, 650, 700, 750, 800, 1000 nm) to capture the spectral dependence of horizontal photon transport over a wide range of molecular scattering. At 1000 nm, molecular scattering is negligible and water vapor absorption is small; cloud absorption is negligible for all wavelengths. For pixels dominated by ice clouds, the scattering phase function and single scattering albedo were used from the general habit mixture of the ice cloud bulk models developed by Baum et al. (2011) (parameterized by the effective radius). For liquid water clouds (minority of cloud pixels), single scattering albedo and asymmetry parameter from Mie calculations were used in conjunction with a Henyey–Greenstein phase function (which generally simplifies irradiance calculations). In this study, all calculations were performed for an ocean surface albedo (Coddington et al., 2010) and for a solar zenith angle of 35° for consistency with the earlier publication by Schmidt et al. (2010). The solar azimuth angle was 60° (northeast). The scene parameters (solar geometry, surface albedo, cloud properties) will be generalized in future work. For each wavelength, 10^11^ (10^12^) photons were used for the sub-scene (large-scale) cloud field, which corresponds to 7 × 10^6^ (4 × 10^6^) per pixel, respectively. MCARaTS was run in the forward irradiance mode with periodic boundary conditions. For each 3-D model run, calculations were also performed using the independent pixel approximation (IPA) where horizontal photon transport is deactivated.

## 5 Relationship between cloud spatial structure, net horizontal photon transport, and its spectral dependence

This section discusses the relationship between spatial structure and spectrally dependent horizontal photon transport based on the small sub-scene. Since true absorption, *A_λ_*, is negligible, *H_λ_* is equal to *V_λ_*, the vertical flux divergence of an inhomogeneous cloud layer as defined in Sect. 2, with *h*_top_ ≈ 13 km and *h*_base_ ≈ 8 km.

Table 1 shows the optical thickness and effective radius for the eight highlighted pixels from Fig. 1 along with *H*_0_, the horizontal photon transport at *λ* = 500 nm, expressed in percent of the incident irradiance. Positive values of *H*_0_ are related to net photon loss to other pixels (“radiation donors”), negative values to net photon gain (“radiation recipient” pixels). In the small domain, values as high as 50% and as low as −125% were attained. When *H*_0_ falls below −100 %, the radiation received through the sides of a column or voxel exceeds that from the top of the domain. Table 1 is sorted by *H*_0_ rather than by optical thickness. It shows immediately that there is no relationship between the optical thickness (or cloud reflectance) and horizontal photon transport. For example, pixel no. 6 is a net radiation donor, whereas pixel no. 4 with roughly the same optical thickness is a net recipient. For the extreme case of zero cloud optical thickness, the effect of horizontal photon transport had previously been observed as clear-sky radiance enhancement in the vicinity of clouds (Wen et al., 2007; Kassianov and Ovtchinnikov, 2008; Várnai and Marshak, 2009; Marshak et al., 2014). Statistically, this enhancement is a function of the distance of a pixel to the nearest cloud. However, the horizontal scale of this dependence varies with the spatial context. Consequently, the distance to a certain cloud element cannot generally be used to parameterize 3-D cloud effects for individual pixels, whether cloud-free or cloud-covered. This is illustrated when considering pixels no. 4–8 in the anvil outflow, which have low optical thickness (around 10) compared to the convective core (optical thickness ≥ 40) overflown from 15:45–15:48 UTC. The small contrasts in optical thickness (reflectance) between the pixels in close proximity tend to drive the sign of *H*_0_ to a greater extent than the exchange of radiation with the (bright) core (for example, no. 6→7, no. 5→4, no. 7→8, but not no. 5→6). On the other hand, pixels no. 2 and 3 have relatively low values of *H*_0_ although they have the largest optical thickness of all eight pixels. While still donors, the magnitude of net horizontal flux to other pixels seems to be diminished by the vicinity to the convective core. Overall, the direction, let alone the magnitude of net horizontal flux, is difficult to predict from the distribution of optical thickness, emphasizing 3-D effects as a non-local phenomenon.

**Figure 3:**
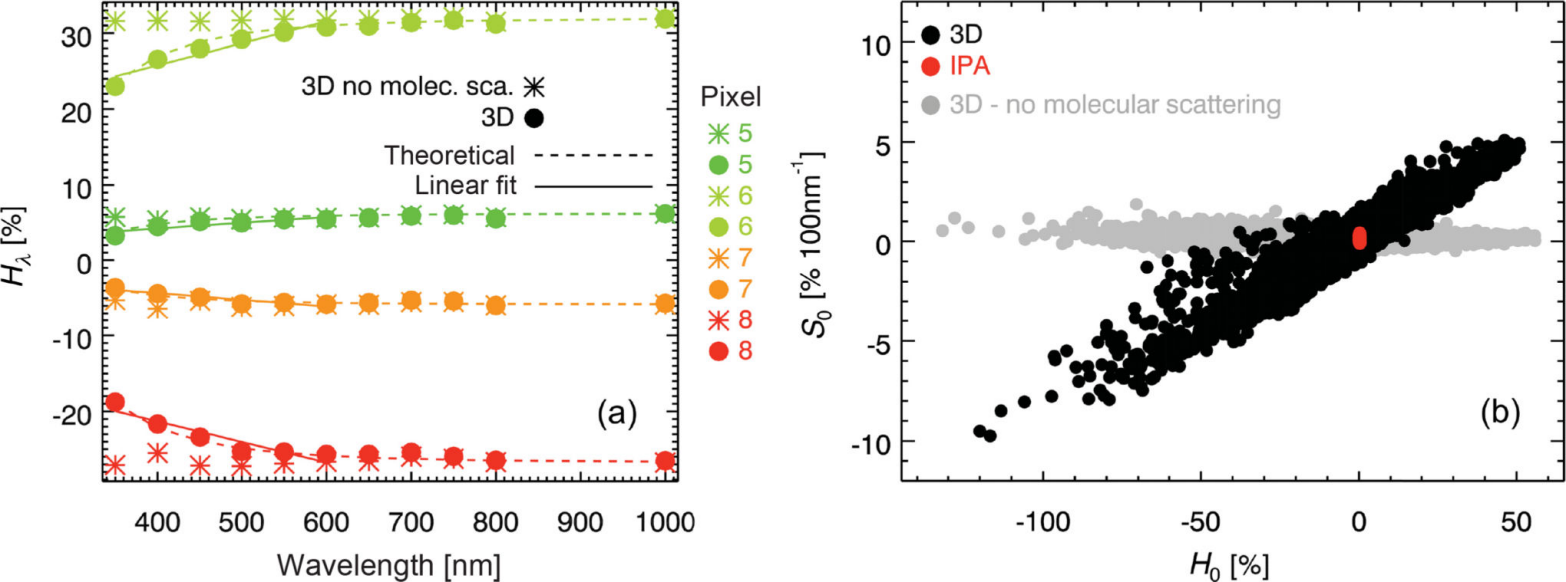
**(a)** The *H_λ_* spectra of pixels {5, 6, 7, 8} from Fig. 1 and Table 1 with (●) and without (*) molecular scattering in the 3-D calculations, as well as a fit based on Eq. (12) from Sect. 6 (dashed lines) and the simplified linear fit for obtaining *S*_0_ (solid lines). **(b)** Spectral slope (*S*_0_) vs. net horizontal photon transport (*H*_0_) from **(a)** (both at 500 nm) for all the pixels from Fig. 1. Only 3-D calculations with molecular scattering (black dots) show the systematic correlation between *H*_0_ and *S*_0_. Disabling molecular scattering (gray dots) incorrectly predicts a spectrally neutral (flat) *H_λ_* (*S*_0_ ≈ 0 for all pixels). By definition, 1-D calculations (IPA, red dots) do not reproduce net horizontal photon transport at all (*H*_0_ = 0 for all pixels).

For the highlighted pixels in Table 1 (no. 5–8), Fig. 3a shows the spectral shape of *H_λ_*. The absolute value *H_λ_* increases with wavelength until it reaches an asymptotic value towards near-infrared wavelengths, which we denote *H*_∞_. Donor pixels (*H_λ_* > 0) are associated with a positive spectral slope, *S_λ_* ≡ d*H_λ_* / d*λ* > 0; recipient pixels have a negative spectral slope. Remote sensing studies (e.g., Marshak et al., 2008; Várnai and Marshak, 2009) had previously established that the above-mentioned radiance enhancement for clear-sky pixels near clouds was associated with “apparent bluing,” and proposed molecular scattering as the underlying cause for this spectral dependence. To demonstrate that the same effect is at work here, molecular scattering was deactivated in MCARaTS, keeping everything else the same in the calculations. In the resulting spectra (* symbols in Fig. 3a), the wavelength dependence in the near-ultraviolet and visible range disappears almost entirely, suggesting molecular scattering as the primary cause for the spectral shape not only for clear-sky, but also for cloudy pixels. This begs the question (addressed in the next section) of how it is possible to observe such a significant spectral effect for cloudy pixels, given that cloud scattering outweighs molecular scattering by far. After turning molecular scattering off, the remaining variability in *H_λ_* is due to the weak dependence of cloud scattering properties on wavelength and droplet or crystal effective radius, as well as minor gas absorption features. Note that the earlier study by Schmidt et al. (2010) remained inconclusive as to the mechanism of the spectral dependence they observed.

To first order, the spectral shape over the range of 350 to 650 nm can be characterized by a single number – the spectral slope at *λ* = 500 nm, *S*_0_ (obtained from a linear fit to *H_λ_* = 350–600 nm, included in Fig. 3a). Table 1 lists the value of *S*_0_ for the eight pixels from Fig. 1, whereas Fig. 3b depicts the relationship between *H*_0_ and *S*_0_ for every pixel. It shows that not only the sign, but also the magnitude of the net horizontal photon transport, is surprisingly well correlated with its slope at 500 nm (in %(100 nm)^−1^). This suggests that the phenomenon observed by Schmidt et al. (2010) for a few isolated data points is a general occurrence throughout a heterogeneous cloud field. The close relationship between the magnitude and spectral shape of net horizontal photon transport is the basis for the spectral parameterization of *H_λ_*, developed in the next section.

In *H*_0_–*S*_0_ space, all IPA calculations (red dots in Fig. 3b) are reduced to the origin because they do not allow horizontal pixel-to-pixel radiation exchange by definition. Owing to periodic boundary conditions, the domain average of *H*_0_ is zero. The calculations without molecular scattering (gray dots) confirm that molecular scattering dominates the spectral shape throughout the domain. The vertical spread of the gray data points is due to the other factors mentioned above (e.g., variability in cloud microphysics). To some extent, it is also apparent in the IPA calculations.

## 6 Physical mechanism and parameterization

Our interpretation of Fig. 3 is that *H_λ_* can be understood as the combination of two terms:

(7) $$H_{\lambda }=H_{\infty }+\delta (\lambda ).$$

**Figure 4:**
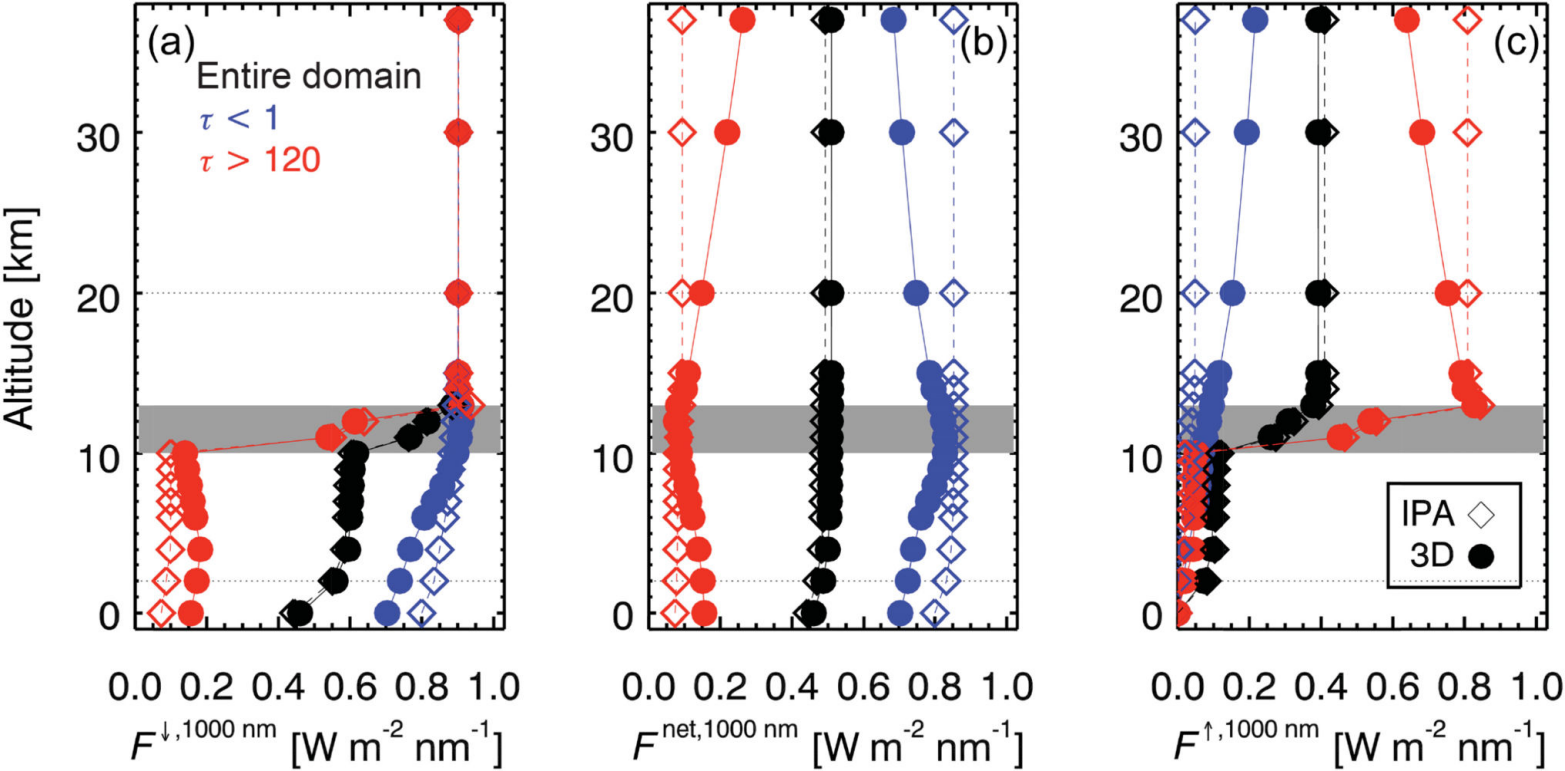
Profiles of **(a)** downwelling, **(b)** net, and **(c)** upwelling irradiance at 1000 nm for the cloud field from Fig. 1. The location of the cloud layer is marked in gray. Both IPA (dashed line, hollow symbols) and 3-D calculations (solid line, full symbols) are shown, averaged over the full domain (black), over all columns with *τ* < 1 (blue), and over columns with *τ* > 120 (red).

**Figure 5:**
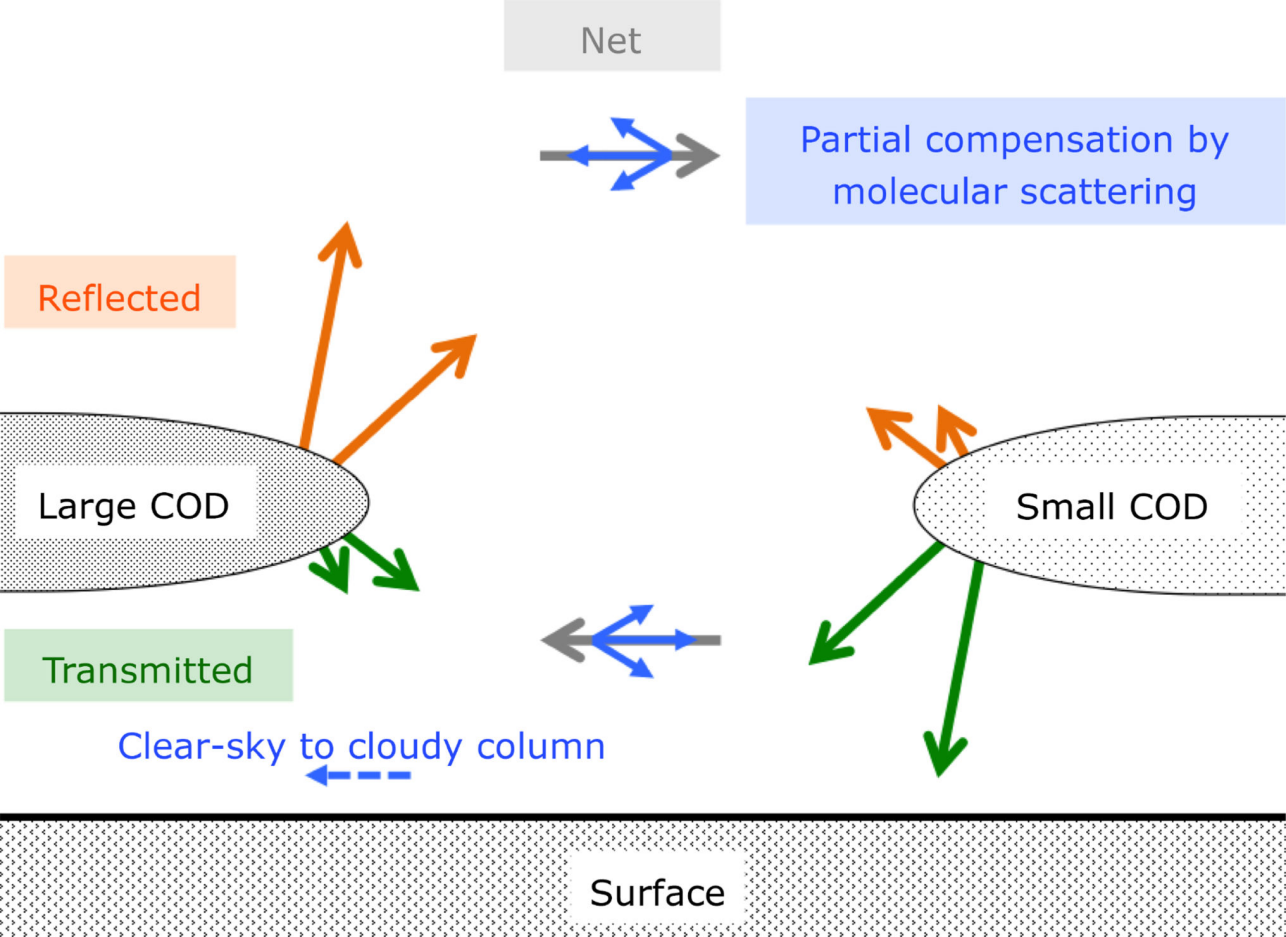
Conceptual visualization of the mechanism of horizontal photon transport.

1. The constant offset *H*_∞_ is caused by column-to-column radiation exchange between cloud elements. This is illustrated by Fig. 4, which shows the vertical profile of (a) downwelling, (b) net, and (c) upwelling irradiance at 1000 nm wavelength for the cloud field from Fig. 1. A change of net irradiance between altitudes *z*_0_ and *z*_1_ corresponds to net radiation loss or gain within that layer. In this case, the domain-averaged profile of net irradiance (black line in Fig. 4b) decreases slightly near the surface, due to small absorption in the wing of the 936 nm water vapor band. When subsampling over columns with a cloud optical thickness *τ* < 1, or *τ* > 120, the 3-D calculations differ from the IPA calculations because column-to-column radiation transfer is enabled. Above the cloud field, columns with high cloud optical thickness have higher reflectance than the domain average (Fig. 4c), and collectively lose radiation to those with lower optical thickness; the opposite is true below the cloud where columns with high optical thickness have lower transmittance (Fig. 4a). The magnitude of the net horizontal photon transport (the difference of net irradiances at the bottom and top altitude of a layer) increases with the geometrical layer thickness. Fig. 5 conceptually depicts the processes at work. Above clouds, net horizontal photon transport (reflected radiance, projected into a horizontal plane) occurs from the high- to low-reflectance column. Below clouds, the direction is reversed because the transmittance of thin clouds is larger than that of thicker clouds. Note that below *τ* ≈ 4, directly transmitted radiation dominates the downwelling irradiance, and the cloud may not act as a “diffuser” as shown in Fig. 5. The direction of the green arrows is then along the direct beam. This simplified figure should not be interpreted to suggest that the net horizontal transport generally occurs along gradients of cloud optical thickness. As stated above, its direction and magnitude depend not only on directly adjacent columns, but also on the large-scale context, which is why a parameterization of 3-D cloud effects in clear-sky areas in terms of the distance to the nearest cloud is only possible in a statistical way, but not on an individual pixel basis (Wen et al., 2007). The value of *H*_∞_ can be obtained from *H_λ_* for wavelengths where molecular scattering becomes negligible and where cloud and gas absorption are small compared to *H_λ_*: *A_λ_* ≪ *H_λ_*. For the purpose of this study, we chose *λ* = 1000 nm: *H*_∞_ ≈ *H_λ_* = 1000 nm.
2. The spectral perturbation *δ_λ_*, superimposed on *H*_∞_, introduces the wavelength dependence of *H_λ_*. It is perhaps not immediately intuitive why molecular scattering would reduce the magnitude of *H_λ_* as indicated by the symbolic blue arrows in Fig. 5. Molecular scattering essentially reduces the directionality of horizontal photon transport by redistributing radiation, part of which can then be detected as enhanced clear-sky reflectance of clouds (Marshak et al., 2008). A different, secondary process occurs when radiation is scattered out of the direct beam in clear-sky areas into cloud shadows (dashed blue arrow in Fig. 5). It is spectrally dependent as *δ_λ_* but, unlike *δ_λ_*, independent of *H*_∞_ and its direction – thus increasing the net radiation under both optically thick and thin clouds. Below 550 nm wavelengths (not shown in Fig. 4), the net irradiance does indeed increase towards the surface, both for *τ* > 120 and for *τ* < 1. This secondary effect is not explicitly captured by the first-order parameterization given below.

We express the proportionality of *δ_λ_* to *H*_∞_ as

(8) $$\delta (\lambda )=-\varepsilon {\left(\frac{\lambda }{\lambda_{0}}\right)}^{-x}H_{\infty }(\varepsilon \geq 0,\lambda_{0}=500\text{ nm}),$$

where (*λ/λ*_0_)^−*x*^ describes the wavelength dependence, and *ε* is the constant of proportionality. The layer thickness for which *H_λ_* is derived affects both *H*_∞_ and *δ_λ_*, but only marginally changes the correlation between them. Therefore, *ε* is a general parameter that can be used for relating spatial inhomogeneities and spectral signature of a cloud scene as a whole. It depends on scene parameters such as surface albedo, solar zenith angle, and cloud micro- and macrophysics (including vertical structure). This dependence is explored in a separate publication (Song, 2016). Using Eq. (8), the spectral slope *S*_0_ can be derived as

(9) $$S_{0}={\frac{dH_{\lambda }}{d\lambda }|}_{\lambda =\lambda_{0}}={\frac{d\delta (\lambda )}{d\lambda }|}_{\lambda =\lambda_{0}}=x\varepsilon \frac{H_{\infty }}{\lambda_{0}}.$$

**Figure 6:**
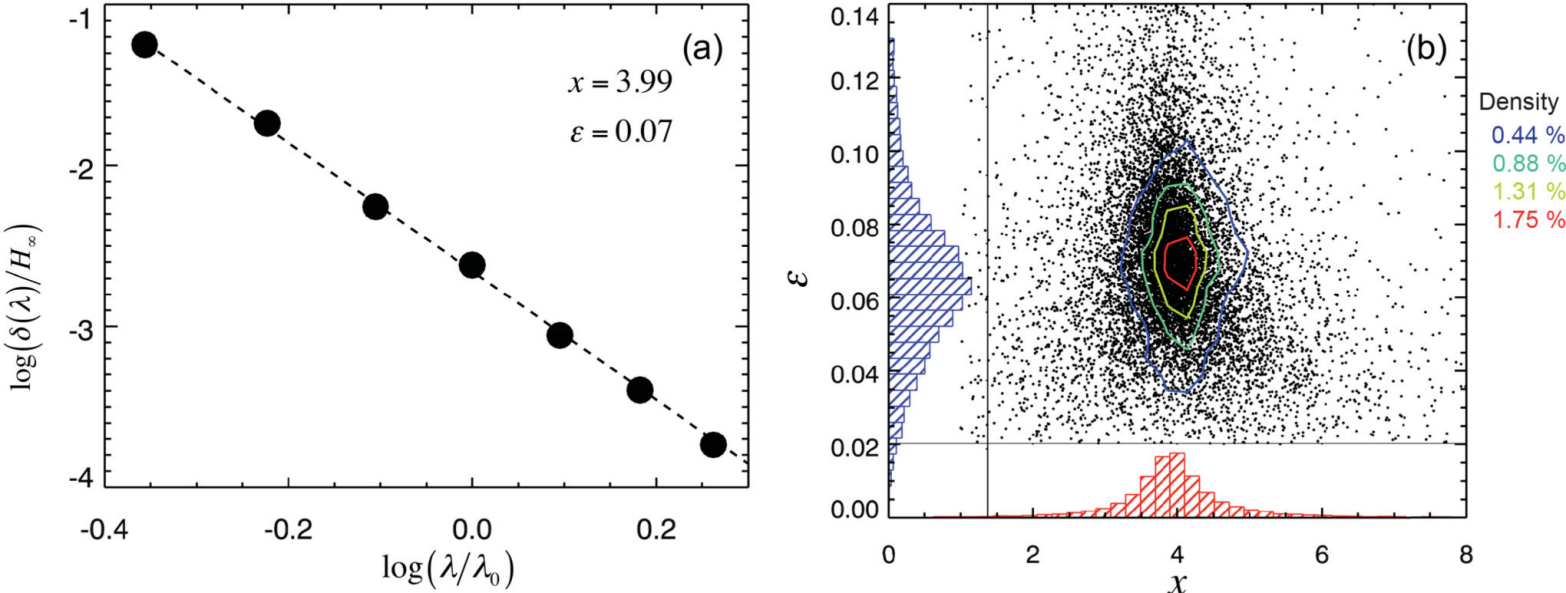
**(a)** An example of the linear regression between $\text{log}\frac{\delta (\lambda )}{H_{\infty }}$ and $\text{log}\frac{\lambda }{\lambda_{0}}$, from which the values of *x* and *ε* can be derived. **(b)** The scatter plot of *x* vs. *ε* for all pixels, joint PDFs *p*(*x,ε*) (contours) as well as the marginal PDFs *p*(*x*) and *p*(*ε*) (histograms). The peak of *p*(*x,ε*), and thus the most likely {*x, ε*} values for the cloud field, is located at {3.85, 0.065}, and the domain-averaged values are {3.91, 0.070}.

**Figure 7:**
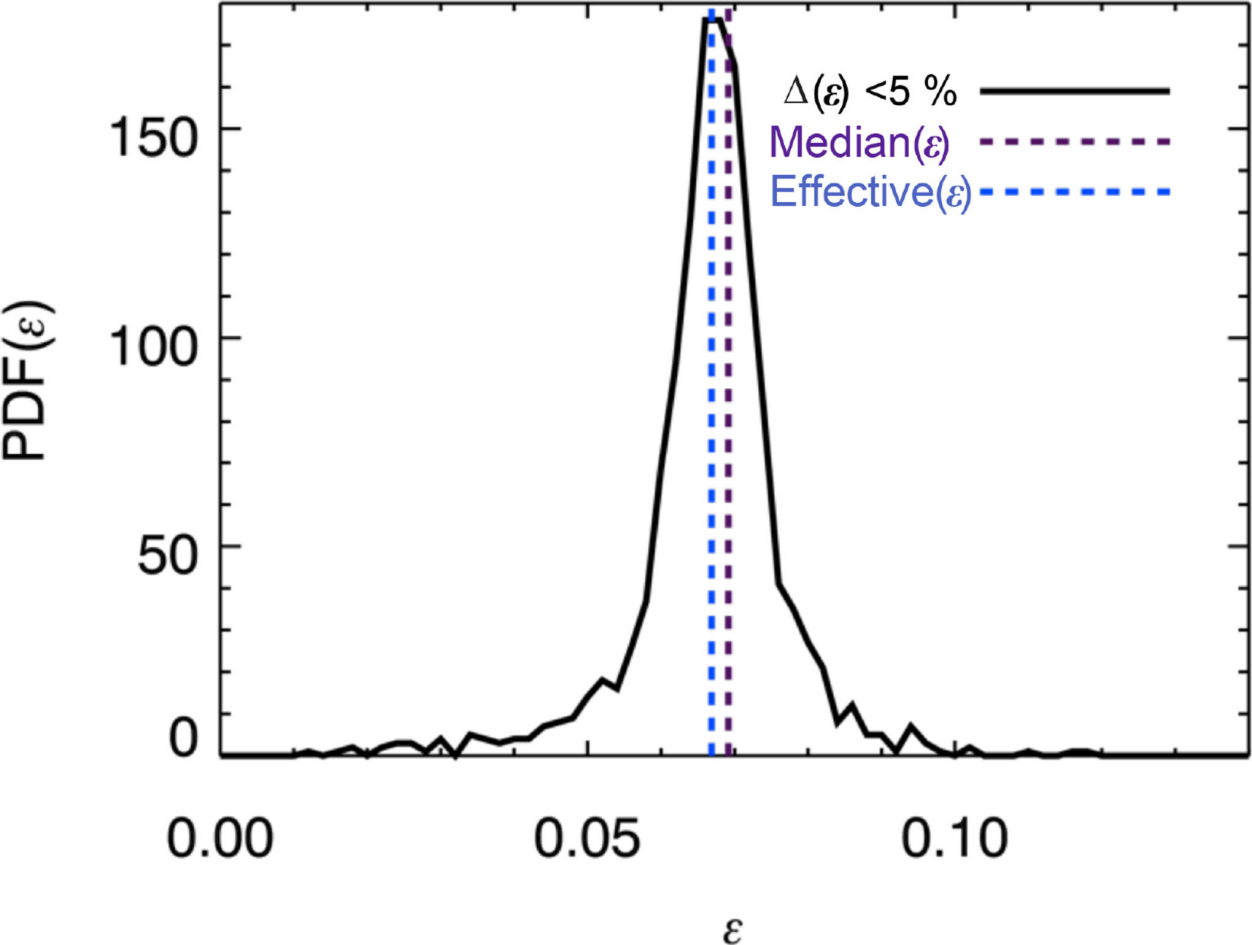
PDF of *ε* for all pixels with Δ(*ε*) < 5 %, median (purple dashed line), and domain-wide effective *ε* derived from regression of *S*_0_ vs. *H*_0_ (blue dashed line).

By combining Eqs. (7) and (8), one obtains *H*_0_ = *H_λ_* = 500 nm = *H*_∞_(1 − *ε*), and Eq. (9) can be rewritten as

(10) $$S_{0}=\frac{x\varepsilon }{1-\varepsilon }\frac{H_{0}}{\lambda_{0}},$$

where *xε*/(1 − *ε*)λ_0_ is the slope of the linear regression derived using all pixels in the cloud domain (for example, in Fig. 3b). Alternatively, one can derive both *ε* and *x* for each individual pixel from the regression of

(11) $$\text{log }(-\frac{\delta (\lambda )}{H_{\infty }})=\text{log }\varepsilon -x\text{ log}\frac{\lambda }{\lambda_{0}},$$

with log *ε* as intercept and *x* as slope, as shown in Fig. 6a. In this example, the fit parameter *x* is about 4 as would be expected for molecular scattering as the underlying physical mechanism. The 2-D probability distribution function (PDF) *p*(*x, ε*) for the population of pixels in the domain peaks at {*x, ε*} ≈ {3.85, 0.065} but has a considerable spread in both parameters, which is caused by pixels with negligible horizontal photon transport (and consequently large uncertainties in the fit parameters). The dashed lines in Fig. 3a show the fitted spectra (labeled “theoretical”) from this approach. For practical purposes, we fix *x* ≡ 4 for the remainder of this paper. This allows

(12) $$H_{\lambda }=H_{\infty }(1-\varepsilon {\left(\frac{\lambda }{\lambda_{0}}\right)}^{-4})$$

to be used instead of Eq. (11) and *ε* and *H*_∞_ to be derived for each pixel from a linear regression of *H_λ_* vs. (*λ/λ*_0_)^−4^ (i.e., *H*_∞_ is no longer a required input parameter as for the logarithmic regression).With *ε* known, *S*_0_ can be calculated from Eq. (9). This is more accurate than the derivation of the slope from a linear fit to the spectrum as used for Fig. 3, which, due to the nonlinearity of the spectral dependence, differs from that of the tangent if finite wavelength intervals are used. The domain-wide “effective” *ε* can then be derived from the slope of the regression line of *S*_0_ vs. *H*_0_ for all pixels (Eq. (10) with *x* = 4). Fig. 7 shows the distribution of *ε* as derived from (12) for all those pixels with an uncertainty of Δ(*ε*) < 5 %. The median of this distribution (0.069) is almost identical to the effective value of *ε* (0.067). The standard deviation of the distribution is about 0.01. This means that the parameterized correlation between net horizontal transport and its spectral dependence can be applied to the domain as a whole as well as for individual pixels; if the spectral shape of *H_λ_* is known, one can infer its magnitude throughout the near-ultraviolet and visible wavelength range. The correlation is robust regardless of the cloud context of a pixel, which is remarkable given the considerable variability in distance-based measures of 3-D cloud effects (Várnai and Marshak, 2009).

Although our study was instigated by aircraft measurements, its findings are also relevant for satellite-based derivations of cloud radiative effects since the spectral perturbations *δ_λ_* propagate into observed radiances (Song et al., 2016). This may be exploited in future applications for deriving correction terms for 3-D radiative effects via their spectral signature.

The mean albedo of an inhomogeneous cloud field derived from CERES observations should be fairly insensitive to 3-D effects because they are statistically folded into anisotropy models of such scene types (if these empirical models adequately accomplish the radiance-to-irradiance conversion for a range of sun-sensor geometries). By contrast, surface cloud radiative effects are much less constrained by direct CERES observations because cloud transmittance has to be derived from concomitant imagery. This is where biases introduced by *H_λ_* are most significant. For the remainder of this paper, we therefore analyze the significance of *H* for varying degrees of spatial aggregation (Sect. 7), and make the connection to cloud transmittance (Sect. 8).

## 7 Scale dependence and spatial aggregation

**Figure 8:**
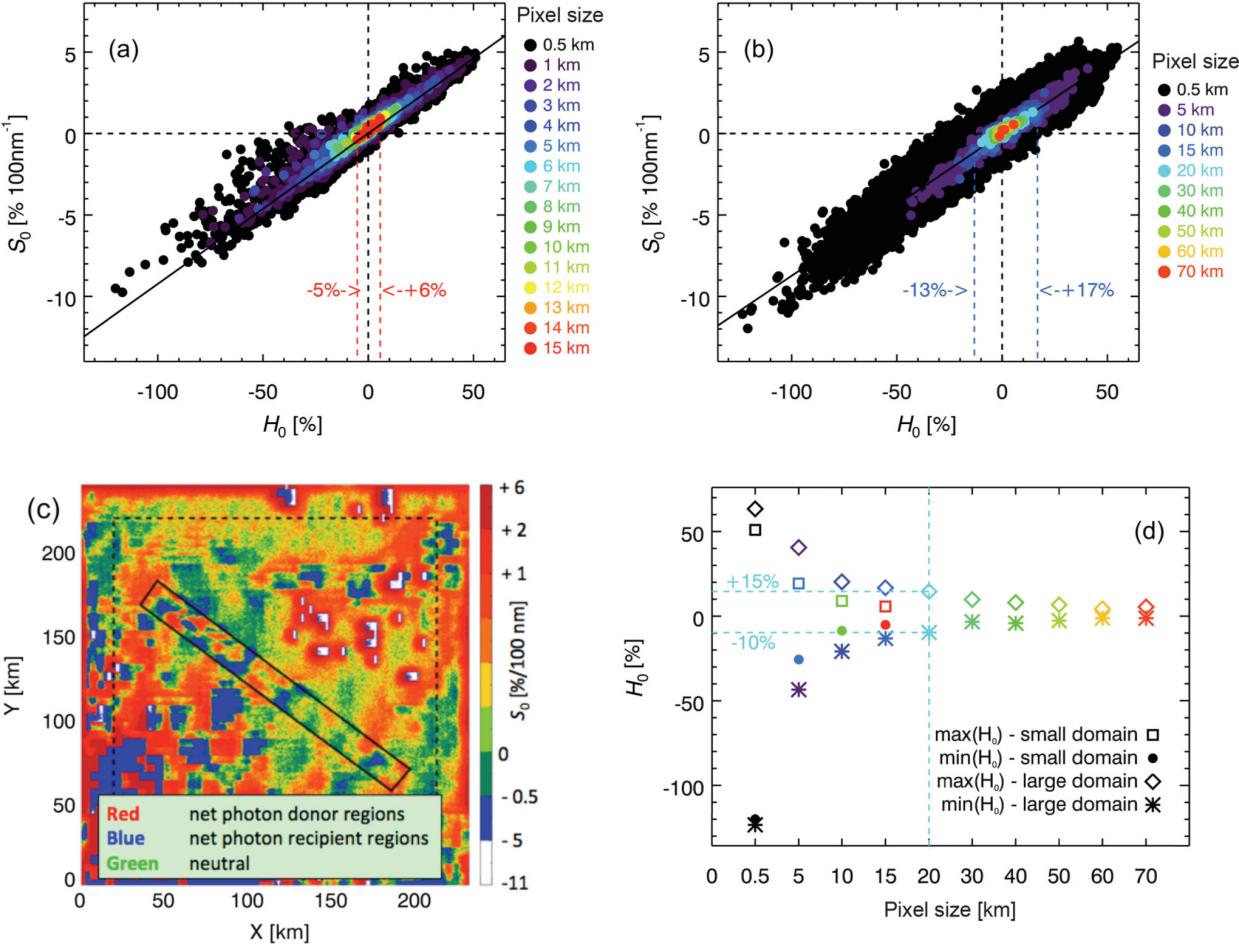
Scatter plot of *S*_0_ vs. *H*_0_ as obtained from linear regression of Eq. (12) for **(a)** the small domain from Fig. 1 and **(b)** the large-scale domain from Fig. 2, spatially aggregated to different scales, including the 20 km super-pixels as highlighted in Fig. 2 (red squares). The dashed lines indicate the range for 15 km pixels. **(c)** Spatial distribution of *S*_0_ from **(b)**. Red (blue) indicates net photon donor (recipient) pixels, and green “neutral zones” (*H_λ_* ≈ *S*_0_ ≈ 0). **(d)** Dependence of max(*H*) and min(*H*) on spatial aggregation scale (km). The color is the same as in **(b)**.

The results presented so far (e.g., in Fig. 3b) are based on calculations at a resolution of 0.5 km. The question is whether the correlation between the magnitude and spectral shape of *H* is scale-invariant, and to what extent the effect of horizontal photon transport can be mitigated by spatial aggregation. To answer this question, we successively coarsened the pixel resolution to 15 km, the largest “super-pixel” contained within the MAS swath (Fig. 1). Figure 8a shows that the correlation is indeed independent of the spatial aggregation scale and thus pixel size. The magnitude of *H*_0_ decreases with pixel size: it ranges from +6 to −5% at 15 km resolution (close to CERES for nadir viewing), compared to about ±50% at 1–5 km (resolution of various MODIS level-2 products). Here, we use the large cloud scene (Fig. 2) to estimate for which aggregation scale beyond 15 km the magnitude of *H*_0_ drops below the radiometric uncertainty of typical space- or ground-based radiometers (3–5 %), at which point 3-D cloud effects become insignificant from a practical point of view.

The results for the large scene, shown in Fig. 8b, confirm that the correlation is preserved for scales up to 70 km. However, *H*_0_ at 15 km resolution varies from +17 to −13% throughout the large-scene domain, much more than in the MAS-only domain (+6 to −5 %). One explanation for this larger range is the greater complexity of the large domain, providing a more extensive sample of cloud variability than the smaller sub-scene. This becomes quite clear when looking at the spatial distribution of horizontal photon transport (Fig. 8c). We chose to plot *S*_0_ (*y* axis in Fig. 8b) rather than *H*_0_; they are practically interchangeable thanks to the correlation between the two. The distribution of effective donor, recipient, and “neutral” regions (red, blue, green, respectively) bears almost no resemblance to the optical thickness field from Fig. 2. This demonstrates once again that horizontal photon transport cannot be derived from the spatial distribution of clouds in any simple way; strong contrasts between negative and positive *H*_0_ (or *S*_0_) can arise in optically thin boundary layer clouds (southwest corner of Fig. 2 and 8c) as well as in optically thick areas (deep convection, northeast corner of cloud scene). Considering the GOES-MAS large-scene results within the boundaries of the MAS swath only (marked by the rectangle in Fig. 8c) allows the net exchange of radiation between the MAS domain and its large-scene context to be estimated. The average value of *H*_0_ within the small-scene subset is +7.9 %, which means that the small scene effectively loses photons to its surroundings. This would not be detectable for such a large aggregation scale (where the entire MAS domain represents a single super-pixel). This net energy export is not reproduced by the calculations based on the MAS-only domain where the mean value of *H*_0_ is zero, in keeping with energy conservation that is satisfied by periodic boundary conditions in the radiative transfer model. The range of *H*_0_ in the MAS-only sub-scene of the GOES-MAS scene is +17 to −6% at 15 km aggregation scale. This is still a larger range than obtained from the MAS-only calculations (+6 to −5 %), even after subsetting the results from the large scene to the boundaries of the small ones. The reason is simply that the 15 km super-pixel size is already half the width of the MAS-only domain. Boundary conditions enforce the convergence of *H*_0_ to zero as the area ratio of pixel to domain size approaches 1, which causes an underestimation of the variability of *H*_0_ for large aggregation scales. By contrast, photons can also travel outside the confines of the domain in the real world as represented by the larger GOES-MAS cloud scene in our study.

This is illustrated in Fig. 8d, which shows the range of *H*_0_ for both the large and the small cloud scene as a function of aggregation scale. At small scales, the range is comparable for the small and large scene. At 15 km aggregation scale, the range obtained from the small scene has decreased to about half that of the large one. At 50 km pixel resolution, *H*_0_ ranges from +7 to −3% (+5 to −1% at 70 km). It is likely that the boundary conditions imposed on the large domain also cause an underestimation of the *H*_0_ variability at these large scales. Nevertheless, these results suggest that above 60 km super-pixel size (about 3 × 3 CERES nadir footprints), horizontal photon transport can be neglected for this cloud scene, based on a 3% uncertainty threshold. This is only true when aggregating all native-resolution pixels, regardless of whether they are flagged as clear sky or as cloud-covered. However, sampling cloudy and clear pixels separately would result in much larger biases than 3% because high optical thickness pixels are more likely to be effective photon donors than low-optical thickness or clear pixels, causing an asymmetry in the distribution of *H*_0_ (Song et al., 2016).

## 8 Significance for cloud radiative effects

In this section, we evaluate the ramifications of net horizontal photon transport on estimates of cloud radiative effects. For any atmospheric column, *H* is connected to *R* and *T* through Eq. (1) and manifests itself in a transmittance and reflectance bias (*λ* index omitted):

(13a) $$\Delta T=T^{\text{IPA}}-T^{3-D}$$

(13b) $$\Delta R=R^{\text{IPA}}-R^{3-D}.$$

Juxtaposing energy conservation for a horizontally homogeneous atmosphere (*T*^IPA^ + *R*^IPA^ = 1) with Eq. (1) for conservative scattering (*A* = 0, therefore *T*^3-D^ + *R*^3-D^ = 1 − *H*) yields the plausible relationship

(14) H=ΔT+ΔR,

which means that the error introduced by horizontal photon transport is partitioned into transmittance and reflectance bias. Since the bias Δ*R* is folded into the empirical radiance-to-irradiance conversion employed by CERES, we focus on Δ*T* in this study.

**Figure 9:**
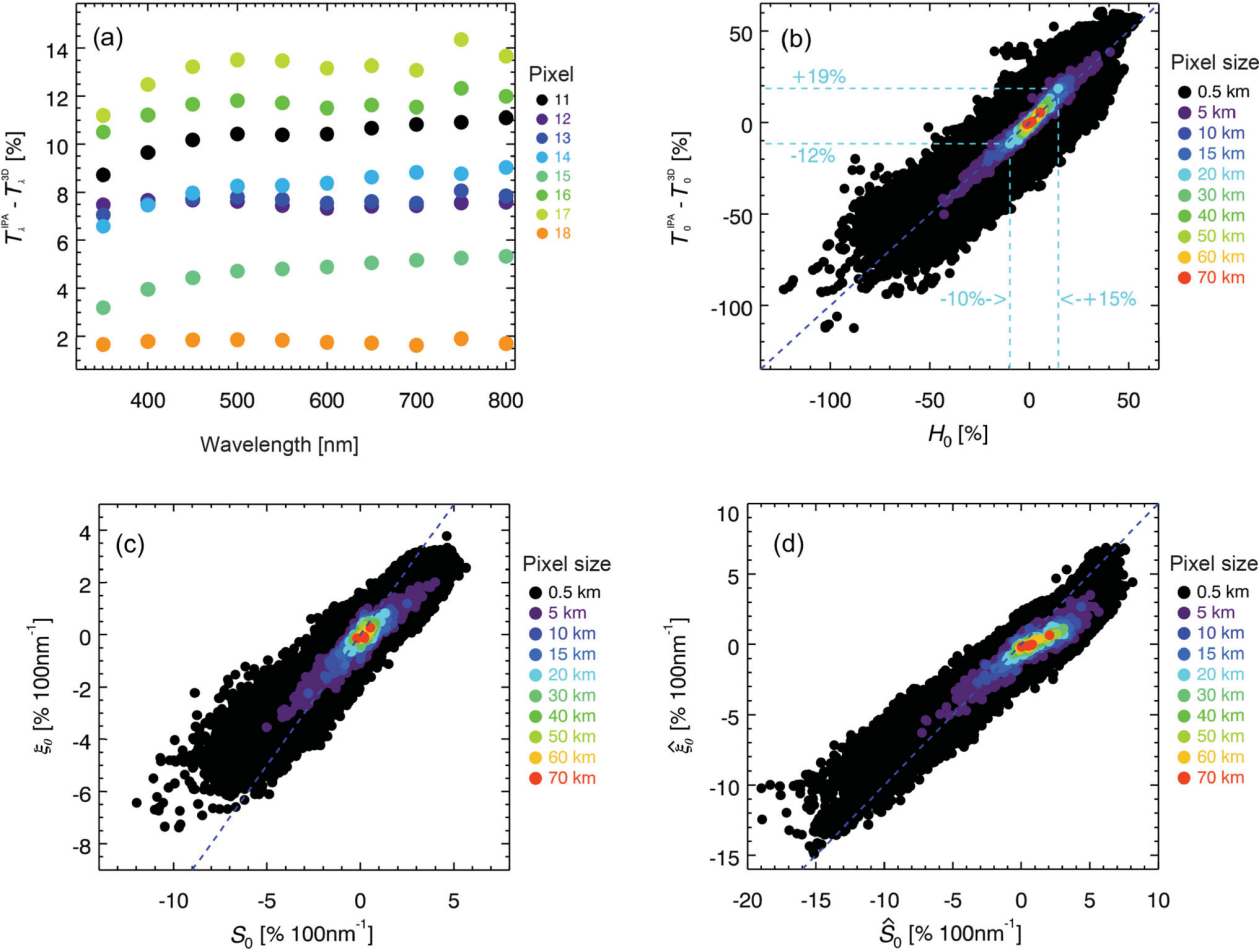
**(a)** Transmittance biases (IPA-3-D transmittance) for the eight super-pixels from Fig. 2. **(b)** Correlation between net horizontal photon transport from Fig. 8b and transmittance bias for multiple spatial aggregation scales. The dashed lines indicate the range of variability for 20 km super-pixel size. **(c)** Correlation of the slopes of the quantities from **(b)**. **(d)** Same as **(c)**, but for a bracket from the surface to cloud top, rather than the cloud layer only.

**Figure 10:**
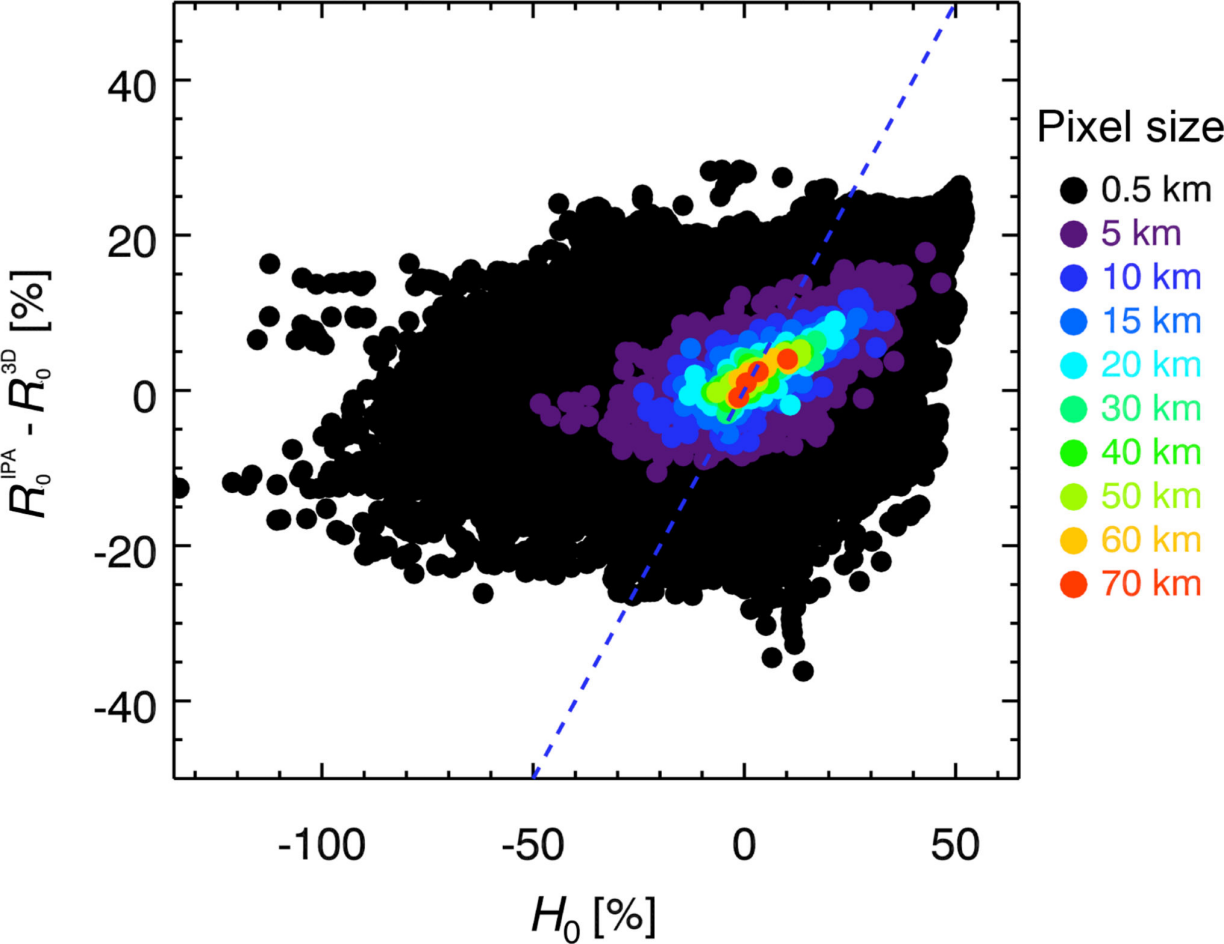
*H*_0_ is only weakly correlated with reflectance biases Δ*R*_0_ (IPA-3-D reflectance) at scales below 15 km, which means that, statistically, biases introduced by horizontal photon transport propagate primarily into transmittance, not albedo. This changes for larger scales.

For the eight super-pixels no. 11–18 from Fig. 2, Fig. 9a shows the IPA bias Δ*T*, ranging from +2 to +14% in the mid-visible spectrum. Its spectral dependence is more complicated than the one shown for *H* in Fig. 3a, with a less obvious correlation between magnitude and spectral shape. Nevertheless, Fig. 9b shows a remarkable correlation between *H*_0_ and Δ*T*_0_ (*T*^IPA^ −*T*^3-D^ at 500 nm) for the same aggregation scales as in Fig. 8b. For example, the *H*_0_ range of +15 to −10% translates into +19 to −12% in Δ*T*_0_ for a horizontal resolution of 20 km. Linear regression between *H*_0_ and Δ*T*_0_ suggests that in this case, *H*_0_ propagates mainly into Δ*T*_0_, whereas it is uncorrelated with Δ*R*_0_ for scales below 20 km (Fig. 10).

For simplicity, the spectral dependence of Δ*T* as shown in Fig. 9a is approximated by

(15) $$\Delta T_{\lambda }=T_{\lambda }^{\text{IPA}}-T_{\lambda }^{3-D}=\xi_{0}{|}_{350-600\text{ nm}}\times (\lambda -\lambda_{0})\;+(T_{0}^{\text{IPA}}-T_{0}^{3-D});\lambda_{0}=500\text{ nm},$$

where *ξ*_0_ is the spectral slope of $T_{\lambda }^{\text{IPA}}-T_{\lambda }^{3-D}$ calculated from the spectrum between 350 and 600 nm. Fig. 9c shows that the spectral slopes of *H* and Δ*T, S*_0_ and *ξ*_0_, are correlated despite the more complicated spectral dependence of *T* compared to that of *H* (Fig. 9a). However, there is clearly no 1 : 1 relationship as found between *H*_0_ and Δ*T*_0_ above. For example, *S*_0_ = −10 % (100 nm)^−1^ corresponds to only *ξ*_0_ = −6 % (100 nm)^−1^. This changes when extending the vertical layer boundaries (8–13 km so far, bracketing only the cloud layer itself) to the atmosphere reaching from the ground to cloud top (Fig. 9d). This distinction is indicated by carets above all quantities. This is slightly different from the definition of *T̂* in Sect. 2 where the upper boundary is the top of atmosphere, not the top of the cloud. The spectral dependencies of *Ĥ* and Δ*T̂* have similar magnitudes (Fig. 9d), as opposed to the equivalent quantities shown in Fig. 9c. However, the relationship between *Ŝ*_0_ and *ξ̂*_0_ is not scale-invariant above 15 km. This means that the vertical bracket for deriving *T, R*, and *H* has to be chosen with consideration of the vertical location of the cloud layer. By contrast, the correlation between *H* and *S* as discussed in Sect. 6 is fairly independent of the layer boundaries and scale.

For future studies of IPA-3-D biases in satellite-derived estimates of surface cloud radiative effects, Fig. 4b suggests the center of a cloud as upper boundary of the bracket where |d*F*_net_/d*z*| reaches a domain-wide minimum because 3-D effects can be vertically separated into a transmittance and reflectance part below and above this level, respectively. Moreover, the correlation between Δ*T* and its spectral dependence *ξ*_0_ (not shown) can be exploited to detect IPA-3-D biases in ground-based irradiance measurements below cloud fields (Song, 2016). While our study suggests that horizontal photon transport mainly propagates into transmittance biases, there is some indication (Fig. 10) that at scales above 20 km, nonzero values of *H*_0_ translate into albedo (reflected irradiance) biases as well. This increasing correlation with scale is probably associated with the gradual decorrelation between *Ŝ*_0_ and *ξ̂*_0_ observed in Fig. 9d. In order to improve satellite-based estimates of cloud radiative effects, it is important to understand how *H*_0_ is partitioned into Δ*T* and Δ*R* (Eq. 14) at different aggregation scales. A detailed study would need to be conducted for different cloud morphologies, sun angles, and surface albedos, and is left for the future.

## 9 Summary and conclusions

Deriving the radiative effects of inhomogeneous cloud scenes from observations by satellite, aircraft, or at the surface is often portrayed as an intractable problem because it cannot be accomplished by isolating a pixel from its spatial context. At the core of the issue is pixel-to-pixel exchange of radiation, or net horizontal photon transport, which occurs over a range of scales. The original motivation for this study was to gain a physical understanding of this phenomenon’s spectral dependence in the near-ultraviolet and visible wavelength range, which had been found in aircraft irradiance observations (Schmidt et al., 2010). We were able to identify molecular scattering as the underlying mechanism for the spectral dependence using 3-D radiative transfer calculations with cloud imagery and radar observations as input. When deactivating molecular scattering in the radiative transfer model, the wavelength dependence disappeared almost entirely in the vertical flux divergence *V*, which comprises net horizontal flux density *H* as well as true layer absorption *A*. To simplify the analysis, we limited our study to conservative scattering by choosing wavelengths with negligible gas or cloud absorption (*A* ≈ 0), and by excluding aerosols. When activated in the model, molecular scattering manifested itself as a spectral perturbation (more accurately: modulation) *δ_λ_* to an otherwise spectrally neutral horizontal flux density *H*_∞_, which in turn could be traced back to horizontal exchange of radiation due to spatial inhomogeneity of cloud elements within the domain. Beyond the original scope of this study, we made a few surprising discoveries:

1. The spectral perturbation *δ_λ_* is not independent of the spectrally neutral part *H*_∞_ caused by the clouds themselves. Instead, the mid-visible spectral slope of *H_λ_* is correlated with *H* itself (i.e., with the magnitude of the spectrally neutral part *H*_∞_), which led to the simple parameterization
  $$\delta_{\lambda }=-\varepsilon {\left(\frac{\lambda }{\lambda_{0}}\right)}^{-x}H_{\infty }.$$
2. We were able to show that the exponent *x* is close to 4, which further confirmed molecular scattering as the dominating physical mechanism behind the spectral perturbation. The constant of proportionality, *ε*, can be regarded as universally valid for all pixels within the cloud domain, independently of the vertical or horizontal spatial distribution of clouds. This means that the spectrally dependent horizontal photon transport can be represented as
  $$H_{\lambda }=H_{\infty }+\delta_{\lambda }=H_{\infty }(1-\varepsilon {\left(\frac{\lambda }{\lambda_{0}}\right)}^{-4})$$
  for each pixel within the domain with *ε* = 0.07 ± 0.01 for the scene we studied. It seems remarkable that one single value of *ε* should suffice to describe the relationship between the magnitude of *H* (caused by clouds) and its spectral dependence (imprinted on *H* by a completely different physical process, molecular scattering) – especially considering the range of different clouds within the domain. The correlation holds for each pixel, no matter what its spatial context may be. Once *ε* is established for a given cloud scene, the spectral perturbations associated with horizontal photon transport can be derived for each pixel if the value of *H*_0_ is known. Conversely, if the spectral shape of *H_λ_* is known at one wavelength, its magnitude can easily be inferred for the whole spectrum. This may be especially significant considering that *H* cannot be directly observed from space. It is likely that the spectral perturbations will propagate into the observed radiances. Indeed, Song et al. (2016) found evidence of this connection in aircraft data, which had previously been reported by Várnai and Marshak (2009) in clear-sky satellite observations near clouds. The close correlation that we found in our study may be a future pathway to inferring the magnitude of *H* from its spectral manifestation in the observed radiances.
3. The correlation and parameterization hold for a range of spatial aggregation scales, and are fairly independent of the location of the bracketing altitudes that define the layer. This scale invariance only breaks down when extending a layer very close to the surface where a secondary spectral effect has to be factored in (see Sect. 6 and dashed arrow in Fig. 5).
4. The observed correlation between *H* and its spectral shape can also be found between transmitted irradiance *T* and its spectral shape, although it is not scale-invariant beyond 20 km.
5. *H* is correlated with Δ*T*, the IPA transmittance bias for each pixel, but not with Δ*R* (at least at small scales). This means that 3-D cloud effects in the form of horizontal photon transport translate almost exclusively into a transmittance bias. At scales above 20 km, a correlation between *H* and Δ*R* does emerge, which requires further study. The correlation between *H* and Δ*T* can potentially be exploited for ground-based spectral irradiance observations (Song, 2016).

Few of these findings could be expected at the outset of our research, and they evoke a number of new questions:

1. How does the discovered correlation and the constant of proportionality in its parameterization, *ε*, depend on scene parameters such as solar zenith and azimuth angle, surface albedo (magnitude and spectral dependence), and cloud morphology and microphysics? What “drives” the parameter *ε*?
2. Can the spectral perturbations associated with *H* indeed be detected in reflected radiances, and can they be used to infer the magnitude of *H* indirectly?
3. Can the findings for the near-ultraviolet and visible wavelength range be generalized to the near-infrared wavelength range where clouds and atmospheric gases do absorb?
4. What are the implications of our findings for estimating aerosol radiative effects (such as heating rates) in the presence of inhomogeneous cloud fields?
5. Can the method by Ackerman and Cox (1981) to correct for horizontal photon transport in aircraft measurements of atmospheric absorption by using a visible channel as basis for the correction of near-infrared absorption be upheld for future measurements, even in the modified form proposed by Kassianov and Kogan (2002)?
6. Can *H* and Δ*T* be derived from spectral perturbations in transmitted irradiance observations by ground-based spectrometers?

Question 2 will be partially addressed by Song et al. (2016); questions 1, 3, 5, and 6 are discussed by Song (2016), and will be further investigated in future publications. Furthermore, questions 3 and 4 are the subjects of active research in the framework of ongoing or planned field missions (NASA ORACLES and CAMP^2^Ex). This publication constitutes a further contribution to the emerging field of cloud-aerosol spectroscopy (Schmidt and Pilewskie, 2012), which is expected to improve the estimation of cloud-aerosol parameters and their radiative effects through spectrally resolved observations from the ground, aircraft, and, ultimately, space.

## 10 Data availability

The MAS (King et al., 2007) and GOES (Heidinger et al., 2007; Walther et al., 2010) level-2 data (the input for the cloud fields) can be obtained at http://lasp.colorado.edu/lisird/resources/lasp/nnx14ap72g/MASL2_07919_09_20070717_1519_1534_V03.hdf and http://lasp.colorado.edu/lisird/resources/lasp/nnx14ap72g/goes12_2007_198_1515.level2.hdf, respectively. More recent versions of the MAS data can be downloaded from https://ladsweb.nascom.nasa.gov/archive/MAS_eMAS/TC4/ (flight 07_919). All other data, the derived 3-D cloud fields, and the irradiance calculations can be requested from the corresponding author.

## Figures and Tables

**Table 1:** Cloud optical thickness *τ*, effective radius *r*_e_, and values of *H*_0_ and *S*_0_ for the eight pixels highlighted in Fig. 1 (sorted by *H*_0_). For pixels 5, 6, 7, and 8, Fig. 3a shows the spectral shape of *H_λ_*.

Pixel	*τ*	*r*_e_ (µm)	*H*_0_ (%)	*S*_0_ (% (100 nm)^−1^)
6	10.3	27.5	28.92	2.36
1	13.0	30.1	21.17	1.56
3	21.2	30.0	13.04	1.08
2	18.1	30.6	9.92	1.63
5	12.2	27.5	4.95	0.48
7	8.0	27.8	−5.18	−0.78
4	11.8	28.2	−18.7	−1.54
8	7.7	24.2	−24.13	−2.46
